# A Patient With Myelin Oligodendrocyte Glycoprotein Positive Encephalitis With Ring Enhancing Lesions on Magnetic Resonance Imaging (MRI) After COVID-19 Exposure

**DOI:** 10.7759/cureus.31844

**Published:** 2022-11-23

**Authors:** Jamie Jacobs, Priscilla Vu, Antonio K Liu

**Affiliations:** 1 Internal Medicine, Adventist Health White Memorial, Los Angeles, USA; 2 Neurology, Adventist Health White Memorial, Los Angeles, USA; 3 Neurology, Loma Linda University School of Medicine, Loma Linda, USA

**Keywords:** intravenous immunoglobulins (ivig), autoimmune encephalitis, myelin-oligodendrocyte glycoprotein (mog), ring-enhancing brain lesion, covid

## Abstract

Myelin oligodendrocyte glycoprotein (MOG) antibody has been associated with a wide range of neurological diseases, from neuromyelitis optica spectrum disorder to acute disseminated encephalomyelitis. However, MOG positivity with isolated encephalitis has been infrequently reported. MRI findings are usually of the demyelination type. In this case, we report on a patient with COVID-19 exposure who presented with altered mental status and multiple ring-enhancing lesions on MRI mimicking metastatic disease. Due to his unusual MRI findings and presentation, the correct diagnosis was not apparent until MOG antibody results came back positive.

## Introduction

MOG is a glycoprotein on the myelin surface. It plays important roles in cellular adhesion, oligodendrocyte microtubule stability, and mediation of the complement activation cascade. Its location on the outermost part of the myelin sheath makes it highly immunogenic. As a diagnostic tool, MOG IgG was discovered in 2007 but was not widely available until more recently [1].

Myelin oligodendrocyte glycoprotein-associated disorder (MOGAD) is an immune-mediated demyelination disorder that affects the central nervous system. Diagnosis is associated with the detection of MOG antibodies with the cell-based assay. The disorder includes neuromyelitis optica spectrum disorder (optic neuritis and transverse myelitis) (NMOSD), acute disseminated encephalomyelitis (ADEM), and encephalitis [2].

There is a wide range of MRI findings that have been described in MOGAD. Common MRI brain features include infratentorial lesions, deep gray matter lesions, pons or cerebellar involvement, and gadolinium enhancement. Ring-enhancing lesions can be seen in the spinal cord but have not been described frequently in the brain. Typical MRI spine findings include longitudinally extensive sagittal T2 lesions in more than three segments, involvement of the conus, and at least two noncontiguous lesions, with some extension to the posterior medulla/area postrema region. Ring shape gadolinium enhancement has been rarely described [3,4].

In this case, we report on a patient who lived among COVID-19-positive family members and a couple of weeks later developed altered mental status without any focal findings. The initial brain MRI was negative; a repeat MRI a few weeks later subsequently showed multiple ring-enhancing lesions on the T1 sequence. After a long turnaround time, a positive MOG IgG antibody finally pointed us to the correct diagnosis. We highlighted the importance of the association of anti-MOG with atypical MRI findings in a patient with encephalitis of unknown etiology.

## Case presentation

The patient was a 65-year-old previously healthy man not on any prescription medication. He was independent and described as "sharp". He lived with family members that tested positive for COVID-19 four weeks before admission. He had a visit to the Emergency Room for vertigo and nonspecific blurry vision three weeks before admission. His examination was non-focal, and an MRI of the head at that time was negative. He was sent home without admission. At an outpatient neurology clinic, he had an electroencephalogram (EEG) that was positive for intermittent epileptic discharges. He denied a history of seizures.

**Figure 1:**
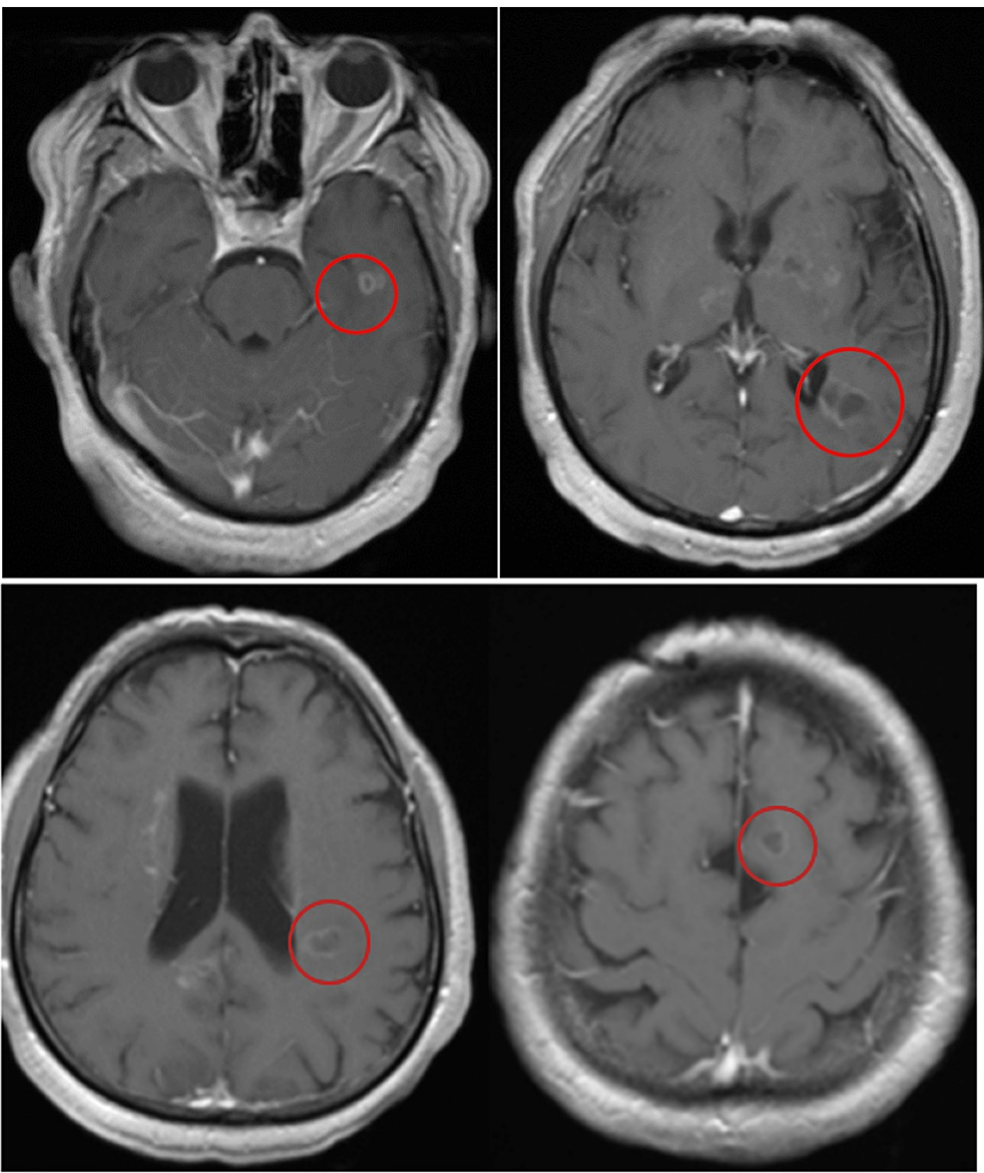
MRI brain T1 sequence showed four ring-shaped post-contrast enhanced lesions (red circles).

During the next three weeks leading up to admission, the patient became increasingly forgetful, confused, and dependent, with even assaultive behavior. He was having visual hallucinations and became delusional. There was no subjective fever or physical illness. On examination upon admission, he was found to be disoriented and incoherent. He was intermittently agitated, combative, and hallucinatory. There were no focal findings in cranial nerve or motor examination. He was strong and required restraints. MRI at this time revealed interval development of multiple FLAIR sequence abnormalities without mass effect. There were four ring-enhancing lesions on the T1 post-contrast sequence (Figure 1). MRI of the whole spine was negative. Cerebral spinal fluid (CSF) examination showed 1/mm3 WBC and protein of 52 mg/dL. The cytological study was negative. His COVID-19 tests were never positive, and his last Pfizer mRNA vaccine booster was five months prior.

Detailed and extensive infectious and oncologic workups were negative, and the patient did not improve. With a high degree of suspicion, the autoimmune neurologic diseases reflexive panel offered by Associated Regional and University Pathologists, Inc. Laboratories (ARUP), Utah, US, was sent. With a turnaround time of 20 days, we were finally alerted to the fact that his MOG IgG titer was 1:20 (normal < 1:10). He was given methylprednisolone and intravenous immunoglobulin for five days. His symptoms and mentation eventually improved. A follow-up MRI showed the resolution of lesions with no more contrast enhancement. He was subsequently discharged home after six weeks of hospitalization. At the time of discharge, he was oriented to name and place and free of hallucination.

## Discussion

In our patient, the etiology for his worsening mentation was never established with systemic workup, brain scanning, and CSF analysis. That was when our attention turned to possible autoimmune encephalitis. The autoimmune neurologic diseases reflexive panel run by ARUP consists of 16 antibodies. MOG antibody was positive in this case, firmly establishing the diagnosis.

MOG IgG positivity has a high specificity for MOGAD, at 97.8%-100%. Clinical features of MOGAD include preceding viral-like prodrome or vaccination, multifocal features of acute disseminated encephalomyelitis, altered mental status, optic neuritis, myelitis, neurogenic bowel or bladder, erectile dysfunction, paraplegia, history of nausea and vomiting and psychiatric manifestations [5]. Cerebrospinal fluid (CSF) findings for MOG-IgG positive encephalitis include mildly elevated white blood cell count (lymphocytic predominant) and elevated protein. Acute symptoms in the majority of patients improve with intravenous methylprednisolone, plasmapheresis, and/or intravenous immunoglobulin. Half of the patients experience a monophasic course. Patients that experience a relapsing course may require long-term immunosuppression [1].

The patient in our case only presented with encephalopathy and altered mental status. His examinations had always been non-focal, free of typical optical or spinal symptoms. A very broad differential diagnosis was present initially. Had this been a MOG IgG negative autoimmune encephalitis, the true diagnosis may have eluded us.

Contrast enhancement has been described in other cases in the literature. In one case, the patient had tumefactive lesions with enhancement. It was reported before the current pandemic, and the patient had numerous physical findings besides encephalopathy [6]. Our patient is different in that lack of other physical findings led physicians to form a much longer differential diagnosis, and the MRI report had referred to those ring-enhancing lesions as metastatic in appearance. The only thing we were sure of was that the lesions were new and acute, as the patient had a negative brain MRI three weeks prior. There is a common feature in that both cases had MRI findings that were reversible mirroring, and clinical improvement with treatment.

Studies have shown that a majority of patients with positive MOG-IgG had prodromal symptoms due to a presumed or confirmed infection, and/or they received the influenza vaccination 1-2 weeks prior. One case of MOGAD occurred two weeks after COVID-19 mRNA vaccination. MOG-IgG was positive, and the lumbar puncture showed oligoclonal bands. MRI brain revealed a right cerebellar peduncle lesion with gadolinium enhancement, which improved slightly after treatment with intravenous methylprednisolone [7].

MOGAD has also been described after COVID-19 infection. In one case, a patient with MOG-IgG positive encephalitis had MRI findings of increased signal intensity in the cortex and basal ganglia with no gadolinium enhancement. The lesions and antibody titers improved after five days of methylprednisolone. Lesions disappeared in 35 days, and the patient made a full recovery [8].

Although COVID-19 infection was never confirmed in our patient, having close contact with multiple family members increased the possibility of acute COVID-19 infection in this patient also. The pathophysiology may involve a mechanism such as molecular mimicry, but more research needs to be done on this matter. We expect it will broaden the spectrum of MOG antibody disease.

## Conclusions

This case is significant because it shows that there is a wide range of MRI findings that may be seen in MOGAD, including ring-enhancing lesions. Patients presenting with confusion or other encephalitis symptoms should be tested for MOG IgG, particularly if they have had a recent COVID-19 infection, exposure, or vaccination. We hope that this case will increase the reader’s suspicion of MOGAD in patients with the altered mental status of unclear etiology and atypical MRI findings because, with appropriate treatment, the majority of patients will improve.
